# KORT – Knee osteoarthrosis radiotherapy trial: Protocol for a multidisciplinary pathway and a planned sham-controlled phase III trial of low-dose radiotherapy in knee osteoarthritis

**DOI:** 10.1016/j.ocarto.2026.100764

**Published:** 2026-02-27

**Authors:** Jörg Andreas Müller, Jassem Alsalloum, Dirk Vordermark, Stefan Delank

**Affiliations:** a Department of Radiation Oncology, University Hospital Halle (Saale), Halle (Saale), D-06120, Germany; b Department of Orthopaedics and Trauma Surgery, Martin-Luther-University Halle-Wittenberg, Halle (Saale), 06120, Germany

**Keywords:** Osteoarthritis, Low dose radiotherapy, Knee, Multidisciplinary phase III trial

## Abstract

**Objective:**

Low-dose radiotherapy (LDRT) is widely used in German-speaking countries for painful degenerative and inflammatory musculoskeletal disorders, but recent sham-controlled trials using non-standard dose concepts reported no superiority over placebo. Methodologically robust trials using established LDRT regimens and guideline-based conservative care are needed. KORT aims to evaluate feasibility and inform a definitive sham-controlled efficacy trial of LDRT in knee osteoarthritis (OA).

**Materials and methods:**

KORT is a two-stage research program at University Hospital Halle (Saale). Stage 1 is a prospective, non-randomized Phase II external pilot comparing (i) standard LDRT alone versus (ii) structured orthopaedic assessment with optimization of guideline-based conservative therapy prior to LDRT. Key feasibility outcomes include recruitment, adherence, data completeness, and follow-up rates. Clinical outcomes include OARSI–OMERACT responder status at 3 months, WOMAC, SF-36, and movement pain (NRS), assessed at baseline, end of treatment, and 3 months; follow-up at 12 and 24 months is planned. Stage 2 is a planned multicenter Phase III randomized, single-blind, sham-controlled superiority trial in which all participants will undergo standardized orthopaedic optimization prior to randomization to LDRT (6 × 0.5 Gy) or sham irradiation.

**Results:**

This protocol specifies eligibility criteria, interventions, outcomes, data management, and the statistical framework. Phase II will provide empirically grounded estimates for workflow refinement and Phase III sample size assumptions.

**Conclusions:**

KORT integrates radiation oncology and orthopedics within a structured multidisciplinary framework and will enable a clinically relevant, adequately powered sham-controlled trial to determine the incremental therapeutic effect of LDRT in knee OA.

## 1 Introduction

Low-dose radiotherapy (LDRT) to treat benign degenerative and inflammatory disorders has been well established in German-speaking countries for decades, particularly among radiation oncologists and increasingly also among orthopaedic specialists. Several thousand patients with painful degenerative joint conditions or enthesiopathies receive this therapy annually, and many experience substantial pain relief [[1], [2], [3], [4], [5]].

Evidence from large observational cohorts supports these clinical experiences. In a multicentre retrospective analysis, Rühle et al. evaluated 970 elderly patients (1185 treated sites) and reported substantial pain reduction in approximately two-thirds of treated regions, assessed by numerical rating scale (NRS) and Pannewitz score, with response rates independent of age (65–74, 75–84, ≥85 years) [6]. Similarly, Hautmann et al. analyzed 295 joints treated exclusively with a linear accelerator and observed durable symptom improvement over 24 months, with median NRS decreasing from 7 at baseline to 3 at follow-up and about one-third of patients reporting minimal or no pain at one year [7]. These findings suggest that LDRT delivered with modern megavoltage equipment achieves outcomes comparable to traditional orthovoltage approaches, although potential advantages of orthovoltage have been proposed for selected clinical scenarios.

Additional support derives from re-irradiation data. In a retrospective analysis of 217 joints receiving a second LDRT course, Hautmann et al. again demonstrated significant and sustained pain reduction up to 24 months, including in patients without an initial response, consistent with the clinical practice of repeating LDRT when symptoms persist or recur [8].

Two randomized, double-blind, placebo-controlled trials from the Netherlands investigated LDRT efficacy for hand osteoarthritis [9] and knee osteoarthritis [10].

In the knee OA trial by Mahler et al., 55 patients aged ≥50 years with ACR-defined knee OA, persistent pain (NRS ≥5), and insufficient response to conservative therapy were randomized to LDRT (6 × 1 Gy) or sham irradiation. At the 3-month primary endpoint, OMERACT–OARSI responder rates were similar (44 % vs. 43 %), and secondary outcomes—including pain, function, and inflammatory markers assessed by ultrasound, MRI, and serum analyses—also showed no between-group differences [10]. The authors concluded that this LDRT regimen did not provide clinically meaningful benefit compared with sham treatment and advised against LDRT for knee OA [10].

Ott et al. subsequently challenged the interpretation of these negative trials, emphasizing methodological limitations including small sample size, short follow-up despite potentially delayed clinical effects, the use of 1 Gy fractions instead of the commonly applied 0.5 Gy regimen, inclusion of patients with long-standing symptoms and advanced OA, and incomplete adjustment for confounders such as BMI or inflammatory activity [11].

The KORT Trial addresses this gap.

## 2 Materials and Methods

### 2.1 Study rationale and overall design

The KORT Trial follows a two-stage design consisting of [1] a prospective, non-randomized Phase II proof-of-concept study and [2] a subsequent randomized, single-blind, sham-controlled Phase III superiority trial.

Phase II evaluates feasibility, workflow, data completeness, and variance estimates to refine the design and sample size of Phase III. The confirmatory Phase III trial will assess the efficacy of LDRT versus sham irradiation following standardized orthopaedic optimization.

### 2.2 Study setting

Both study phases are conducted at the University Hospital Halle (Saale) within a multidisciplinary collaboration between Radiation Oncology and Orthopaedic Surgery. While Phase II is single-center, Phase III will be conducted as a multicentre trial to ensure generalizability and adequate recruitment.

### 2.3 Eligibility criteria

#### 2.3.1 Inclusion criteria

• Age ≥50 years
• Radiologically confirmed knee osteoarthritis (Kellgren–Lawrence grade 1–3)
• Pain intensity NRS <8 at baseline

#### 2.3.2 Exclusion criteria

• Inadequate response to guideline-based conservative therapy (analgesics, physiotherapy, physical modalities)
• Fibromyalgia or suspected somatoform pain disorder
• Knee pain duration >5 years
• Previous RT to the affected knee
• Indication for simultaneous LDRT of other joints or anatomical sites

These criteria aim to ensure a homogeneous population in which the therapeutic effect of LDRT can be appropriately evaluated.

### 2.4 Recruitment and consent

Patients are identified via standard orthopaedic referrals or initial outpatient consultations. Screening follows predefined radiologic and clinical criteria. Eligible patients receive standardized written and verbal information before providing written informed consent. Recruitment patterns, screen failures, and dropouts are documented to assess feasibility for Phase III.

### 2.5 Study arms and allocation (phase II)

In the Phase II proof-of-concept study, allocation to the two study arms is intentionally non-randomized to reflect routine clinical practice and to allow early identification of potentially meaningful differences.

• Standard arm: LDRT without prior orthopaedic optimization
• Optimized arm: Structured orthopaedic consultation including review and adjustment of conservative therapy, followed by LDRT

The comparative results of these two arms will indicate whether orthopaedic optimization should be standardized and mandatory in the subsequent Phase III randomized trial.

### 2.6 Interventions

The interventions evaluated in the KORT Trial reflect the combined multidisciplinary approach that is increasingly used in clinical practice for the management of knee osteoarthritis. In the Phase II proof-of-concept study, participants receive either LDRT alone or undergo a structured orthopaedic optimization prior to LDRT. Based on the results of this preliminary phase, the subsequent Phase III randomized trial will compare active LDRT with sham irradiation, both following a standardized orthopaedic optimization pathway.

### 2.7 Orthopaedic assessment and conservative treatment optimization

Participants allocated to the optimized arm in Phase II undergo a structured orthopaedic consultation prior to initiation of RT. The purpose of this visit is to ensure that each patient receives evidence-based, guideline-compliant conservative therapy according to the German S3 Guideline for knee osteoarthritis before LDRT is considered.

The orthopaedic assessment includes.

• a review of prior diagnostic imaging (and knee radiographs if not yet available),
• a focused physical examination assessing alignment, range of motion, stability, strength, and gait,
• evaluation of previous exercise therapy and non-pharmacological treatments,
• and a structured review of prior pharmacological and intra-articular therapies.

### 2.8 Guideline-based therapeutic optimization

Following this assessment, conservative therapy is adapted using a structured, stepwise, guideline-driven algorithm.

#### 2.8.1 Pharmacological optimization

First-line therapy consists of topical non-steroidal anti-inflammatory drugs (NSAIDs), which are initiated or reinforced whenever appropriate.

If topical NSAIDs are ineffective or unsuitable, oral NSAIDs may be prescribed after reviewing gastrointestinal, renal, hepatic, and cardiovascular risks. Patients at increased gastrointestinal risk receive proton pump inhibitor prophylaxis.

If NSAIDs cannot be used or are insufficient, occasional acetaminophen may be considered for short-term relief.

For stronger pain when other agents are contraindicated or ineffective, metamizole may be used selectively.

Weak opioids are not routinely recommended and are reserved only for rare, short-term use when no other pharmacological option remains appropriate.

#### 2.8.2 Exercise and physiotherapy

Exercise therapy represents a core component of care. Patients receive an individualized, guideline-based program including strength, endurance, and movement training. Supervised sessions are recommended when feasible.

Patients are counseled that initial discomfort may occur but that long-term adherence reduces pain and improves function and quality of life.

Aquatic exercise therapy may be added when indicated, and manual therapy may be used only in combination with exercise.

#### 2.8.3 Mechanical and functional measures

The use of walking aids (e.g., crutches) may be recommended to unload the joint.

Knee orthoses or footwear modifications are not initiated routinely but may be considered when exercise alone is insufficient, when joint instability is present, or when biomechanical abnormalities suggest benefit.

Kinesiotaping may be used selectively under similar conditions but is not part of routine care.

#### 2.8.4 Documentation of prior intra-articular interventions

Intra-articular treatments are not part of the optimization strategy, but their prior use is systematically recorded, including.

• corticosteroid injections,
• hyaluronic acid,
• PPSB injections,
• and prior radiosynoviorthesis, with detailed documentation of type and timing.

In Phase II, this orthopaedic optimization ensures that RT is introduced as part of a standardized and well-defined conservative treatment sequence, allowing more accurate evaluation of treatment response.

In the Phase III trial, this comprehensive guideline-based optimization will be provided uniformly to all participants prior to randomization, ensuring that the subsequent comparison between LDRT and sham irradiation isolates the specific incremental effect of the radiotherapeutic component. The full standardized assessment form used during this visit is provided in Table S1**.**

### 2.9 Low-dose radiotherapy (LDRT)

All participants in the Phase II study receive low-dose radiotherapy following the standard regimen commonly used for benign musculoskeletal disorders in German-speaking countries. Treatment is delivered via a WOMED T-200 device equipped with an MXR-226 (Comet) X-ray tube.

The standard protocol consists of.

• 6 fractions of 0.5 Gy,
• administered over approximately 2–3 weeks,
• targeting the affected knee joint.

A second identical treatment series may be administered after approximately twelve weeks if the patient's clinical response is insufficient. This two-series approach reflects established practice and is supported by clinical experience and mechanistic evidence that anti-inflammatory effects are dose-dependent but optimally induced by low single doses.

Radiotherapy delivery, including patient positioning, field definition, and treatment verification, follows established protocols for benign disease radiotherapy.

### 2.10 Sham irradiation (phase III trial)

In the planned Phase III randomized superiority trial, the comparator intervention will be sham irradiation. Sham treatments will be conducted in the same treatment room with identical procedural steps, including patient positioning, device operation, acoustic signals, and treatment duration. However, no ionizing radiation will be delivered.

**Fig. 1:**
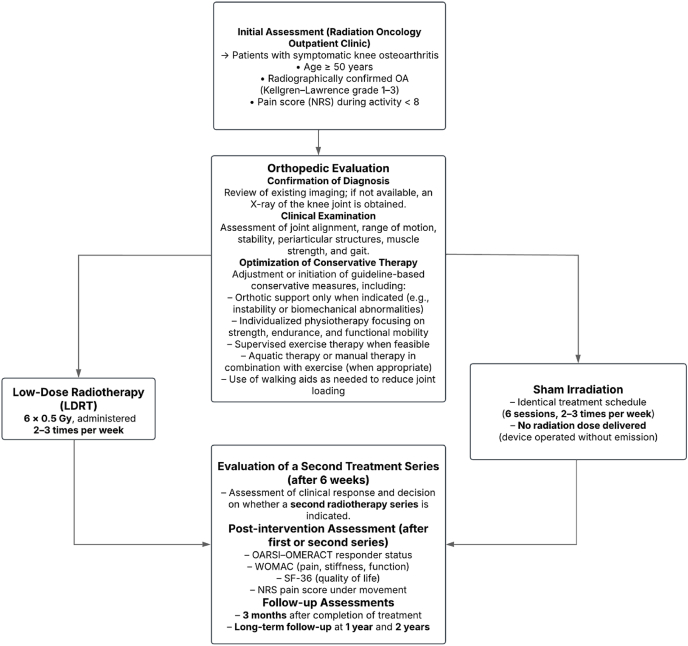
Study flowchart of the planned Phase III sham-controlled KORT Trial.

This approach maintains participant blinding and ensures that any observed differences between the study arms can be attributed to the biological effects of radiation rather than contextual or procedural factors. All participants in the Phase III trial will have undergone standardized orthopaedic optimization prior to randomization, ensuring ethical acceptability of sham treatment and preventing deprivation of recommended conservative therapy. The complete procedural sequence, including orthopaedic optimization, randomization, treatment allocation, and follow-up assessments, is illustrated in the Phase III study flowchart (Fig. 1).

### 2.11 Outcome measures

#### 2.11.1 Primary endpoint

• OARSI–OMERACT responder status at 3 months

#### 2.11.2 Secondary endpoints

• WOMAC (total and subscales: pain, stiffness, function)
• SF-36 health-related quality of life
• Numerical Rating Scale (NRS) for pain

The same endpoints will be used in the Phase III superiority trial.

### 2.12 Data management

Data are recorded electronically using pseudonymized case report forms. Range checks, completeness reviews, and selected double-entry procedures ensure data quality. Adverse events and protocol deviations are documented according to institutional guidelines.

### 2.13 Statistical analysis

The statistical analysis plan for the KORT Trial is structured around the two-phase design of the study, with distinct objectives for the Phase II proof-of-concept study and the subsequent Phase III randomized, single-blind, sham-controlled trial. While the Phase II analysis is exploratory and primarily descriptive, its findings are essential for refining and finalizing the statistical parameters of the Phase III confirmatory study. All analyses will be conducted using the statistical software R.

#### 2.13.1 Analysis of the primary endpoint

The primary endpoint in both phases is the OARSI–OMERACT responder status at 3 months after completion of radiotherapy or sham irradiation.

#### 2.13.2 Phase II (proof-of-concept)

Phase II is descriptive and aims to generate estimates to inform the Phase III design.

Responder rates in the two non-randomized study arms (LDRT alone vs. orthopaedic optimization + LDRT) will be summarised as proportions with 95 % confidence intervals.

Between-arm differences will be expressed as risk differences and risk ratios (95 % CIs).

No formal hypothesis testing will be performed.

Findings will be used to refine assumptions regarding effect size, variance of continuous outcomes, and dropout rates for the Phase III sample size calculation.

#### 2.13.3 Phase III (randomized sham-controlled trial)

In the confirmatory Phase III trial, the primary ITT analysis will compare responder rates between LDRT and sham irradiation using a chi-square test or Fisher's exact test, depending on expected cell counts.

Effect measures (risk ratio, risk difference, odds ratio) will be reported with 95 % confidence intervals.

A per-protocol analysis will be performed as a secondary assessment of robustness.

### 2.14 Analysis of secondary endpoints

Secondary outcomes include the WOMAC (total and subscales), SF-36 (summary and subscales), and NRS pain score.

These outcomes are assessed at baseline, at the end of radiotherapy (or second series), and at 3 months.

#### 2.14.1 Within-group changes

To evaluate how symptoms evolve over time within each treatment arm, changes from baseline to the end of treatment and from baseline to the 3-month follow-up will be analyzed using standard paired statistical procedures. For outcomes that follow an approximately normal distribution, paired t-tests will be applied. If the distributional assumptions for parametric testing are not met, the corresponding non-parametric Wilcoxon signed-rank test will be used instead.

To quantify the magnitude of these within-group changes, effect sizes will be reported alongside the test results. Cohen's *d* will be used for parametric comparisons, while the rank-biserial correlation (*r*) will be presented for non-parametric analyses.

#### 2.14.2 Between-group differences

Comparisons between treatment groups—either between the two Phase II arms or between active LDRT and sham irradiation in Phase III—will be conducted using independent t-tests for normally distributed data or Mann–Whitney U tests for non-normally distributed variables.

Where appropriate, an analysis of covariance adjusting for baseline values will be employed. This approach improves precision by accounting for initial symptom levels and thereby increases statistical power, while also reducing potential bias arising from minor baseline imbalances between study groups.

### 2.15 Longitudinal modeling

If data completeness permits, a mixed-effects repeated-measures model will be applied to evaluate trajectories across all time points.

This model accounts for intra-individual correlation, accommodates missing data under missing-at-random (MAR) assumptions, and allows exploration of time × treatment interactions.

### 2.16 Predefined subgroup analyses

Exploratory subgroup analyses will descriptively evaluate potential effect modifiers, including.

• age (<65 vs. ≥65 years),
• sex,
• BMI categories,
• Kellgren–Lawrence grade.

In Phase III, logistic regression models with interaction terms will be used to explore treatment-by-covariate interactions, yielding adjusted odds ratios with 95 % confidence intervals.

These analyses are exploratory and will not be used for confirmatory claims.

### 2.17 Handling of missing data

To minimize missing data, the 3-month follow-up may be conducted via structured telephone interview.

For multi-item questionnaires (WOMAC, SF-36), missing items will be handled according to the developers’ proration rules.

For the primary endpoint, the main approach will be complete-case analysis.

Sensitivity analyses may include multiple imputation, particularly in the Phase III trial.

### 2.18 Sensitivity analyses

Phase III sensitivity analyses will include.

• intention-to-treat vs. per-protocol comparisons,
• analyses with and without multiple imputation,
• multivariable logistic regression adjusting for relevant baseline covariates.

These analyses will test the robustness of the primary results under alternative assumptions.

### 2.19 Sample size considerations

The sample size planning for the KORT Trial is based on its two-stage design. For the initial Phase II proof-of-concept study, no formal hypothesis-driven calculation is required. Instead, a pragmatic target of 25 patients per study arm (total n = 50) has been chosen. This number is consistent with methodological recommendations for external pilot studies and is expected to provide sufficiently stable estimates of variance, feasibility, and recruitment dynamics while avoiding unnecessary over-recruitment in an exploratory phase.

The formal sample size calculation is relevant for the subsequent Phase III randomized, single-blind, sham-controlled superiority trial. Based on the available literature, a clinically meaningful difference in OMERACT–OARSI responder rates of 15% points between active LDRT and sham irradiation (e.g., 58 % vs. 43 %) was assumed for planning purposes. Using a two-sided significance level of α = 0.05 and a statistical power of 80 %, this results in an estimated requirement of 134 participants per arm, corresponding to a total of 268 participants. If a one-sided test were justified, the required number would decrease to 122 participants per arm (total n = 244).

These figures are considered preliminary. A central purpose of the Phase II study is to obtain empirically grounded estimates of response rates, variability of secondary outcomes, dropout patterns, and operational feasibility. After completion of Phase II, these data will be used to recalculate and refine the final sample size for the Phase III trial in accordance with established recommendations for external pilot studies (Whitehead et al., 2016). This stepwise approach ensures that the definitive trial is appropriately powered without relying solely on assumptions derived from external studies.

### 2.20 Ethical considerations

The study has been approved by the local ethics committee. All participants provide written consent.

## 3 Discussion

Two recent randomized, blinded, sham-controlled trials from the Netherlands—by Mahler et al. in knee osteoarthritis and Minten et al. in hand osteoarthritis—did not demonstrate a measurable clinical benefit of low-dose radiotherapy compared with sham irradiation [9,10]. As emphasized by Ott, Micke et al. [11], several methodological limitations may have contributed to these negative findings, including very small sample sizes, the short 3-month follow-up period despite known delayed LDRT effects, and the inclusion of patients with long-standing and severe symptoms. Both trials also used 1 Gy fractions rather than the established 6 × 0.5 Gy regimen widely applied in German-speaking countries, and did not sufficiently account for clinical factors such as BMI-related inflammation or chronicity of symptoms.

The KORT Trial is designed to address these concerns. It uses guideline-based conservative therapy prior to LDRT, strict eligibility criteria to reduce clinical heterogeneity, and the biologically supported 0.5 Gy fractionation scheme. Long-term follow-up at 1 and 2 years ensures detection of delayed therapeutic effects. The Phase II proof-of-concept study evaluates feasibility, optimizes processes, and refines effect-size assumptions for the subsequent multicenter randomized sham-controlled Phase III trial. This stepwise approach aims to provide the robust evidence base recommended by Ott et al. and others.

The KORT Trial integrates radiation oncology and orthopaedic care within a standardized multidisciplinary framework. It employs the internationally established 6 × 0.5 Gy LDRT regimen, supported by biological data demonstrating stronger anti-inflammatory effects of low single doses. Guideline-based conservative therapy is systematically optimized before LDRT, addressing an important limitation of earlier randomized studies. Strict eligibility criteria limit heterogeneity, improving internal validity. Finally, long-term follow-up at one and two years allows assessment of delayed clinical responses, which are well documented in historical LDRT cohorts.

The Phase II component is non-randomized and single-center, limiting causal inference and external validity. Its sample size is pragmatic and not powered to detect between-group differences. Results may be influenced by referral patterns and local patient characteristics. These limitations are acceptable within the context of an external pilot study. They will be addressed in the planned multicenter Phase III randomized, sham-controlled trial, which will incorporate refined sample size parameters and standardized pre-randomization conservative therapy.

## Abbreviations

***CI***: Confidence interval
***KL***: Kellgren–Lawrence classification
***LDRT***: Low-dose radiotherapy
***MRI***: Magnetic resonance imaging
***NSAIDs***: non-steroidal anti-inflammatory drugs
***NRS***: Numerical Rating Scale
***OA***: Osteoarthritis
***OARSI***: Osteoarthritis Research Society International
***OMERACT***: Outcome Measures in Rheumatology
***PPSB***: Polysulfated polysaccharide (PPSB) injection
***PPI***: Proton pump inhibitor
***RT***: Radiotherapy
***SF-36***: Short Form-36 Health Survey
***VAS***: Visual analogue scale
***WOMAC***: Western Ontario and McMaster Universities Osteoarthritis Index
