# Supplementary material for: KORT – Knee osteoarthrosis radiotherapy trial: Protocol for a multidisciplinary pathway and a planned sham-controlled phase III trial of low-dose radiotherapy in knee osteoarthritis
Source: Osteoarthr Cartil Open. 2026 Feb 27;8(2):100764. doi: 10.1016/j.ocarto.2026.100764 (PMC12996768; doi:10.1016/j.ocarto.2026.100764)
Supplement: Multimedia component 1 [file mmc1.docx]

# Supplement

## Table S1. Standardized Orthopaedic Knee Assessment Form Used in the KORT Trial

## Patient Information

Name: _________________________ Side: ☐ Right ☐ Left ☐ Bilateral

Date of Birth: __________________ Height: ______ cm Weight: ______ kg Occupation: __________________

## Medical History

Duration of symptoms: _________________________________________

Course of symptoms: ☐ Progressive ☐ Non-progressive

History of trauma: ☐ Yes ☐ No If yes, describe: ________________________

Analgesic medication: ☐ Yes ☐ No If yes, specify: _______________________

Pain characteristics: Start-up pain / Night pain / Rest pain / Pain during movement

Pain score (NRS) during movement (0–10): ______

Stair climbing: ☐ Normal ☐ Difficult ☐ Unable

Mechanical blockages: ☐ Yes ☐ No

Walking distance: ☐ Unlimited ☐ Limited to: ______ meters

Previous knee surgeries: ☐ Yes ☐ No If yes: ____________________________

Sports activity: ☐ Yes ☐ No Type: __________________

Physical therapy: ☐ Yes ☐ No Last session: ______ Effect: ______

## Guideline-Based Conservative Treatment

Topical NSAIDs: ☐ Used ☐ Not used

Oral NSAIDs: ☐ Used ☐ Not used

PPI gastroprotection: ☐ Yes ☐ No

Acetaminophen: ☐ Used ☐ Not used

Metamizole: ☐ Used ☐ Not used

Weak opioids (short-term only): ☐ Used ☐ Not used

Exercise therapy: ☐ Supervised ☐ Unsupervised ☐ Not performed

Aquatic exercise therapy: ☐ Yes ☐ No

Manual therapy (with exercise only): ☐ Yes ☐ No

Walking aids: ☐ Yes ☐ No

Knee orthosis/footwear modification: ☐ Indicated ☐ Not indicated

## Intra-Articular Treatments

Corticosteroid injections: ☐ Yes ☐ No Date(s): ______

Hyaluronic acid injections: ☐ Yes ☐ No Date(s): ______

PPSB injections: ☐ Yes ☐ No Date(s): ______

Radiosynoviorthesis: ☐ Yes ☐ No Date(s): ______

**Clinical examination:**

| **Gait** | Stable/ Unstable | Limping: right/ left | Assistive device:  Yes/ No  Type: |
| --- | --- | --- | --- |
| **Leg alignment** | Neutral | Varus: right left  Intermalleolar distance: | Valgus: right left  Intermalleolar distance: |
| **Skin** | Scars: Yes /No | Intact/ Not intact | Joint effusion: Yes/ no  Other findings: |

|  | right | left |
| --- | --- | --- |
| **Range of motion** | Extension/Flexion: / | Extension/Flexion: / |
| **Ligament Stability** | - Anterior drawer test: positive / negative - Posterior drawer test: positive / negative - Pivot shift test: positive / negative - Collateral ligaments: stable / lax - Medial: Grade ______ - Lateral: Grade ______ | - Anterior drawer test: positive / negative - Posterior drawer test: positive / negative - Pivot shift test: positive / negative - Collateral ligaments: stable / lax - Medial: Grade ______ - Lateral: Grade ______ |
| **Meniscus Signs** | - Hyperflexion pain: yes / no (medial / lateral) - Steinmann I: positive / negative (medial / lateral) - Payr sign: positive / negative - McMurray test: positive / negative (medial / lateral) | - Hyperflexion pain: yes / no (medial / lateral) - Steinmann I: positive / negative (medial / lateral) - Payr sign: positive / negative - McMurray test: positive / negative (medial / lateral) |
| **Patella** | - Position: alta / baja - Mobility: ______ - Compression pain: yes / no | - - Position: alta / baja - Mobility: ______ - Compression pain: yes / no |

### **Imaging**

- X-ray of the affected knee joint: __________________________
- Kellgren–Lawrence grade: __________________________
- MRI: __________________________

### **Diagnosis**

### **Recommended Therapy**

### **Included in the Study:** yes / no
